# Effectiveness of High-Protein Energy-Dense Oral Supplements on Patients with Malnutrition Using Morphofunctional Assessment with AI-Assisted Muscle Ultrasonography: A Real-World One-Arm Study

**DOI:** 10.3390/nu16183136

**Published:** 2024-09-17

**Authors:** Juan José López-Gómez, David Primo-Martín, Angela Cebria, Olatz Izaola-Jauregui, Eduardo Jorge Godoy, Paloma Pérez-López, Rebeca Jiménez Sahagún, Beatriz Ramos Bachiller, Jaime González Gutiérrez, Daniel A. De Luis Román

**Affiliations:** 1 Endocrinology and Nutrition Department, Clinical Universitary Hospital of Valladolid, 47003 Valladolid, Spain; 2 Investigation Centre Endocrinology and Nutrition, Faculty of Medicine, University of Valladolid, 47003 Valladolid, Spain; 3 DAWAKO Medtech S.L., Parc Cientific de la Universitat de Valencia, Calle del Catedratic Agustín Escardino Benlloch, 9, 46980 Paterna, Spain; 4 Escola Tècnica Superior d’Enginyeria, Department d’Informàtica, Universitat de València, Avenida de La Universidad, s/n, 46100 Burjassot, Spain

**Keywords:** nutritional support, artificial intelligence, malnutrition, sarcopenia

## Abstract

*Background:* User-friendly tools for assessing nutrition status and interventions in malnourished patients are crucial. This study evaluated the effectiveness of a personalised nutrition intervention using a novel oral nutritional supplement and AI-supported morphofunctional assessment to monitor clinical outcomes in patients with disease-related malnutrition (DRM). *Methods:* This prospective observational study involved patients receiving concentrated high-protein, high-calorie ONS (cHPHC-ONS), per usual clinical practice. Comprehensive assessments were performed at baseline (B_0_) and three months (M3) post-intervention. *Results:* 65 patients participated in the study. Significant decreases were observed in the percentage weight loss from B_0_ (−6.75 ± 7.5%) to M3 (0.5 ± 3.48%) (*p* < 0.01), in the prevalence of malnutrition (B_0_: 93.4%; M3: 78.9%; *p* < 0.01), severe malnutrition (B_0_: 60.7%; M3: 40.3%; *p* < 0.01), and sarcopenia (B_0_: 19.4%; M3: 15.5%; *p* < 0.04). Muscle area increased (*p* = 0.03), and there were changes in the echogenicity of the rectus femoris muscle (*p* = 0.03) from B_0_ to M3. In patients aged ≥60, an increase in muscle thickness (*p* = 0.04), pennation angle (*p* = 0.02), and handgrip strength (*p* = 0.04) was observed. There was a significant reduction in the prevalence of malnutrition (B0: 93.4%; M3: 78.9%; *p* < 0.01) and severe malnutrition (B0: 60.7%; M3: 40.3%; *p* < 0.01). *Conclusions:* In patients with DRM, a personalised intervention with cHPHC-ONS significantly reduces the prevalence of malnutrition, severe malnutrition, and sarcopenia and improves muscle mass and function.

## 1. Introduction

Malnutrition is a highly prevalent and significant health concern among patients with different pathologies. Current data indicate that disease-related malnutrition (DRM) affects nearly 28–73% of hospitalised patients [1]. Across Europe, 34% of patients are estimated to be malnourished or at risk of malnutrition at hospital admission, with a higher prevalence among older adults (39%) [2,3,4]. In outpatient settings, the prevalence of DRM varies widely, from 21% to 69% [4]. In Spain, recent data show that the prevalence of DRM is 29.7% among hospitalised patients according to the Global Leadership Initiative on Malnutrition (GLIM) criteria [5].

DRM can have a profound impact on body composition, particularly muscle mass. Malnutrition significantly reduces muscle mass, resulting in decreased muscle strength and function, impaired physical performance, difficulties in daily activities, and, eventually, sarcopenia [6]. Recent reports showed that sarcopenia is present in 9.7% of patients with DRM [7]. Alongside the consequences of physical impairment (such as falls and fractures), sarcopenia further exacerbates DRM-related complications, including prolonged hospitalisation, poor disease prognosis, higher mortality, and impaired quality of life (QoL) [8,9,10].

Given the profound impact of DRM on the energy–protein requirements and the decreased intake due to the underlying pathology [11], patients with DRM need to adjust the consumption of nutrients to the requirements with an oral diet and, if necessary, with nutritional interventions. Several international guidelines recommended an energy intake of 30 kcal/kg body weight (BW)/day, with a high-quality protein intake of 1.2–1.5 g/kg BW/day [12,13]. The use of hypercaloric and hyperproteic oral nutritional supplements (ONS) is a usual practice to treat malnutrition, with established clinical and economic benefits [14], and it is recommended by many clinical guidelines for DRM [12,13]. However, the selection of the appropriate ONS for patients with malnutrition is crucial, as higher or lower protein intakes may be harmful in specific subgroups of patients with DRM [15].

However, the main challenge with ONS for patients with DRM is compliance. Noncompliance can stem from various factors, including loss of appetite, large volume/portion, treatment complexity, gastrointestinal (GI) intolerance of ONS, long duration of treatment, and monotonous or unsatisfactory taste or texture [4]. Offering a diverse range of flavours in energy-dense nutritional supplements (>2.0 kcal/mL) can enhance patient compliance by catering to patient preferences and reducing the volume required to meet patient’s nutritional needs [16]. Additionally, high-protein supplements with a high whey protein content are related to a better tolerance to the ONS [17]. Furthermore, whey protein is recognised as one of the most effective nutritional sources for promoting muscle mass maintenance and development. Its high availability of amino acids, including a significant amount of leucine, leads to a quick increase in blood amino acid levels, which directly and strongly stimulates protein synthesis in response to feeding [18], making it particularly beneficial for patients with sarcopenia. However, to effectively reduce muscle breakdown between meals and over a longer period of time (6–7 h), it is important to incorporate other types of proteins, such as casein. Due to its slow speed of digestion, casein facilitates sustained amino acid production, resulting in a moderate but more prolonged increase in plasma amino acid concentrations following ingestion [19,20]. Combining fast- and slow-acting proteins can ensure a supply of amino acids that starts shortly after protein ingestion (due to the presence of whey) but is sustained over time (due to the presence of casein), resulting in an improvement in protein synthesis and, most importantly, net protein balance in the body, which is a key factor for muscle growth and retention [21].

In order to address the barriers to the use of ONS in DRM, a concentrated high-protein, high-calorie oral nutritional supplement with a combination of a blend of high-quality proteins (cHPHC-ONS) has been developed. This formula contains high nutritional concentration (≥2.1 kcal/mL; 32g of protein per 200 mL) and combines fast-acting and long-lasting high-quality proteins (60% whey protein; 40% casein). This novel intervention, combined with ensuring that the patient adheres to the prescribed ONS, can improve the nutritional and functional outcomes of patients with DRM.

The evaluation of the effect of treatments in clinical nutrition must be based on changes in body composition and muscle function. Nevertheless, in usual clinical practice, the most used tool is classic anthropometry, which could interfere with many pathological conditions such as inflammation, congestion, or obesity. Nutritional assessment with a morphofunctional point of view helps us diagnose and monitor DRM and sarcopenia more accurately and allows us to adapt our medical nutrition therapy [22]. Recent evidence-based consensuses recommend the use of routinely available tools in clinical practice, such as muscular ultrasonography (US), for morphofunctional assessment of muscle loss in patients with DRM [22]. Novel artificial intelligence (AI)-based software can improve the diagnostic performance of muscle US [23].

In this real-world study, we evaluated the effectiveness of personalised nutritional interventions using cHPHC-ONS in patients with DRM or at high risk of malnutrition and sarcopenia. This study used morphofunctional assessment to help monitor DRM and sarcopenia more accurately and adapt the personalised plans [22].

## 2. Methods

The study was approved by the Medical Research Ethics Committee (CEIm) of the East Valladolid Area (registration code: PI 22-2559). All study procedures adhered to the principles of the Declaration of Helsinki. We obtained signed informed consent from all eligible participants before enrolment.

### 2.1. Study Desing and Eligibility Criteria

The present study was a prospective, observational, single-arm study that included adult (aged ≥18 years) patients who were at risk of malnutrition with a positive Malnutrition Universal Screening Tool (MUST) test [24] and were prescribed personalised nutritional intervention, including cHPHC-ONS, by their treating physician according to usual clinical practice. Patients were recruited from the Endocrinology and Nutrition Service of the East Valladolid Area, Spain, over the period from January 2022 to December 2023. The last follow-up visit was carried out in March 2024. We excluded patients with neurological diseases with inability to walk, chronic kidney disease stage IV or higher, uncontrolled liver disease, and those who refused to sign the informed consent.

The study procedures included nutritional history, history of the underlying disease, anthropometric measures, electric bioimpedanciometry, muscular US, and handgrip strength. The morphofunctional assessment was performed at the beginning of the nutritional intervention and three months later.

### 2.2. Nutritional Intervention

The personalised nutritional plan included both dietary counselling and oral nutritional supplementation. Dietary counselling was provided by a qualified dietitian who offered specific dietary advice and strategies to enhance the nutritional content of regular foods. This individualised approach aimed to enrich patients’ diets using ordinary foods, thereby addressing their unique nutritional needs. In addition to dietary counselling, ONS was implemented using a novel concentrated high-protein, high-calorie formula (cHPHC-ONS). This supplement is a nutritionally complete formula, ready-to-drink liquid, designed to address malnutrition and optimise muscle protein synthesis. It provided ≥2.1 kcal/mL and 32g of protein per 200mL, with a combination of high-quality whey protein (60%) and casein (40%), around 3.4 g of intrinsic leucine per 200 mL (Nestlé Health Science, Vevey, Switzerland) (Supplementary Table S1). The prescription of cHPHC-ONS, in the form of one or two bottles (200 mL) per day and for three months, was determined based on the estimation of the patient’s dietary intake using a semi-quantitative method and their specific nutritional requirement.

### 2.3. Study Variables

We collected the demographic and clinical characteristics of the included patients. Additionally, the nutritional assessment included anthropometric measurements, bioelectrical impedanciometry (BIA), muscular US, handgrip strength, and laboratory findings. The anthropometric measurements included height, weight, Body Mass Index (BMI), arm circumference (AC), and calf circumference (CC). These variables were selected based on the recent evidence and consensuses regarding the evidence-based measurements for assessing malnutrition and sarcopenia [25,26].

The BIA (BIA 101 Nutrilab; EFG Akern, Pisa, Italy) was conducted in the morning after patients had spent 20 min in a supine position and following an overnight fast. This method measured the impedance components, including resistance (Rz), reactance (Xc), and phase angle (PhA), calculated as ([Xc/Rz] × (180º/π) [27]. Both resistance and reactance values were adjusted for the patient’s height. The Appendicular Skeletal Mass Index (ASMI, kg/m^2^) was determined using electrical impedanciometry and calculated with Sergi’s formula [28]. The diagnostic criteria for low muscle mass of the European Working Group on Sarcopenia in Older People (EWGSOP2) were used (ASMI < 7 kg/m^2^ in men and ASMI < 5.5 kg/m^2^ in women) [26].

The muscular US (Mindray Z60, Madrid, Spain) was performed on the rectus femoris (RF) of all subjects due to the ease of RF detection by non-radiology specialists, the reproducibility of its repetition, and the relationship of RF with walking and standing. The procedure was carried out with participants in a supine position using a 10–12 MHz multifrequency linear matrix probe. The US probe was positioned perpendicular to the transverse axis of the dominant leg (lower third of the distance between the iliac crest and the upper border of the patella) [29]. The images obtained through ultrasonography were then processed using an AI-based ultrasound imaging system (PIIXMED^TM^; DAWAKO MedTech; Valencia, Spain).

The PIIXMED^TM^ system facilitates feature extraction in 2D for conventional B-Mode US imaging and can calculate single values per feature for a region of interest (ROI). From the identified features and application of various algorithms, diverse biomarkers were extracted and processed to analyse the ROI’s morphological architecture, muscle quality based on echogenicity, and different matrix-based biomarkers of texture [23]. Variables measured in the muscular US included the RF muscle area (RFMA) in cm^2^, representing the cross-sectional area of the muscle belly within the ROI, and the RF muscle thickness (RFMT) in cm, indicating the muscle thickness of the cross-section of the muscle belly. Additionally, subcutaneous fat (SCFAT) in cm was measured as the thickness of subcutaneous adipose tissue in the longitudinal section, and the pennation angle in degrees was determined as the angle between the muscle fibres and the lower aponeurosis.

**Figure 1:**
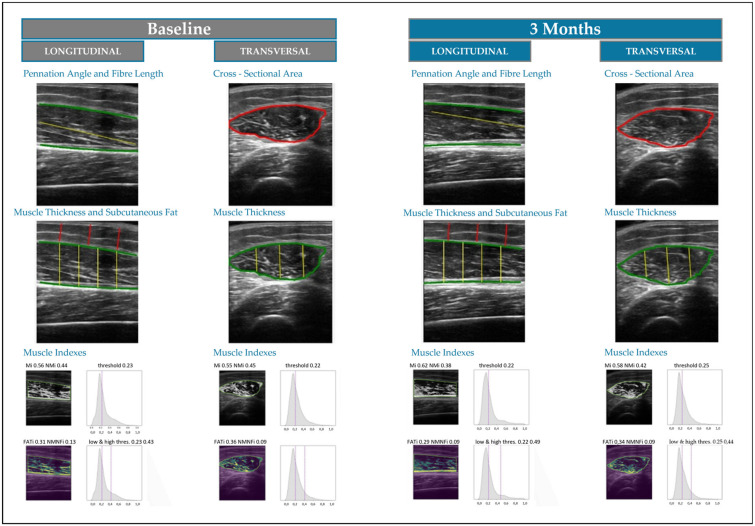
Differences between ultrasonographic variables determined by AI-based ultrasound imaging system PIIXMED^TM^.

Grey level non-uniformity matrix (GLN) was also assessed to quantify the even distribution of grey levels in the image, where higher values indicated a more heterogeneous distribution of grey levels. Muscle quality indexes were determined using the multi-thresholding algorithm, which is based on histogram echogenicity and grey intensity and defines thresholds to separate the pixels of an ultrasound image into different classes. This algorithm determines threshold values for three categories: muscle (Mi index), fat (FATi index), and other complex structures such as collagen, connective tissue, and fibrosis (NMNFi index) [30]. These indexes were represented as percentages (Figure 1).

Muscle functionality was assessed using handgrip strength measured by a JAMAR^®^ dynamometer (Basel, Switzerland). During this test, patients were seated with their arms at a right angle to the forearm and performed dynamometry with their dominant hand. Low muscle strength was defined as <27 kg in men and <16 kg in women, according to the cut-off points proposed by the EWGSOP2 [26]. Finally, the biochemical parameters (Cobas c-711 autoanalyser (Roche Diagnostics, Basel, Switzerland)) included albumin, prealbumin, *C*-reactive protein (CRP), CRP/prealbumin ratio, and creatinine.

### 2.4. Study Endpoints

The primary endpoint of the present study was the percentage of weight loss three months after the administration of ONS. The secondary endpoints included the changes in the BIA parameters, muscular US parameters, compliance with the prescribed doses, and the changes in the prevalence of malnutrition and sarcopenia. Malnutrition was diagnosed according to the GLIM criteria [31], and sarcopenia was diagnosed according to the EWGSOP2 [26].

### 2.5. Statistical Analysis

The intervention analysis was conducted using an intention-to-treat approach. Qualitative variables were presented as percentages (%). The normality of continuous variables was assessed using the Kolmogorov–Smirnov test. Continuous variables were displayed as means ± standard deviation (SD), while non-continuous variables were shown as medians and interquartile range (IQR). Differences between parametric variables were analysed using the Student’s *t*-test for both paired and unpaired data. Non-parametric variables were analysed using the Kruskal–Wallis K-test and the Mann–Whitney U-test. Differences between qualitative variables were examined using the chi-square test. Changes from baseline to three months post-intervention were assessed to determine differences between variables. A *p*-value of less than 0.05 was considered statistically significant. Data analysis was performed using the statistical software package SPSS 23.0 (SPSS Inc., Chicago, IL, USA).

## 3. Results:

### 3.1. Baseline Characteristics of the Included Patients

**Figure 2:**
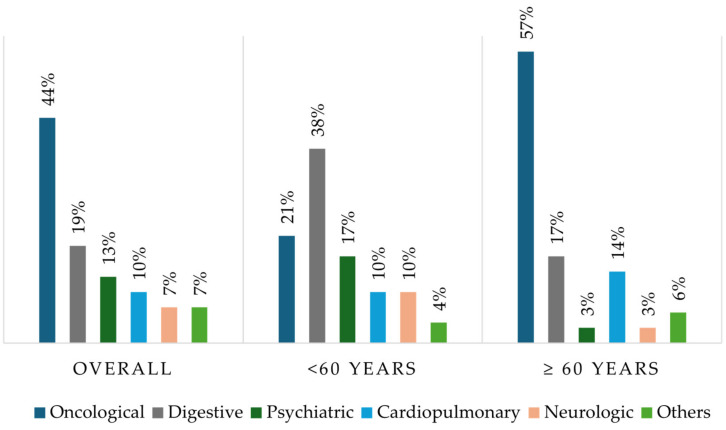
Prevalence of the underlying pathologies in the whole sample and according to the age of the patients.

A total of 65 patients participated in the study, with a female predominance (63.1%). The mean age of the participants was 59.35 ±17.35 years, with 36 patients (55.4%) being over 60 years old. The pathologies that caused DRM are shown in Figure 2. Notably, among patients aged 60 and above, a substantial percentage (57%) had oncological pathologies.

At baseline, 61 (93.4%) patients had malnutrition according to GLIM criteria (with 37 patients (60.7%) classified as having severe malnutrition). Among those over 60 years old, 20 patients (60.6%) were severely malnourished, compared to 17 patients (60.7%) under 60 years old. Additionally, 12 (19.4%) patients were diagnosed with sarcopenia, with eight patients (24.2%) over 60 years old and four patients (13.8%) under 60 years old. Differences in the baseline morphofunctional assessment by sex are presented in Table 1, and differences between patients over 60 years and those under 60 years are shown in Supplementary Table S2.

**Figure 3:**
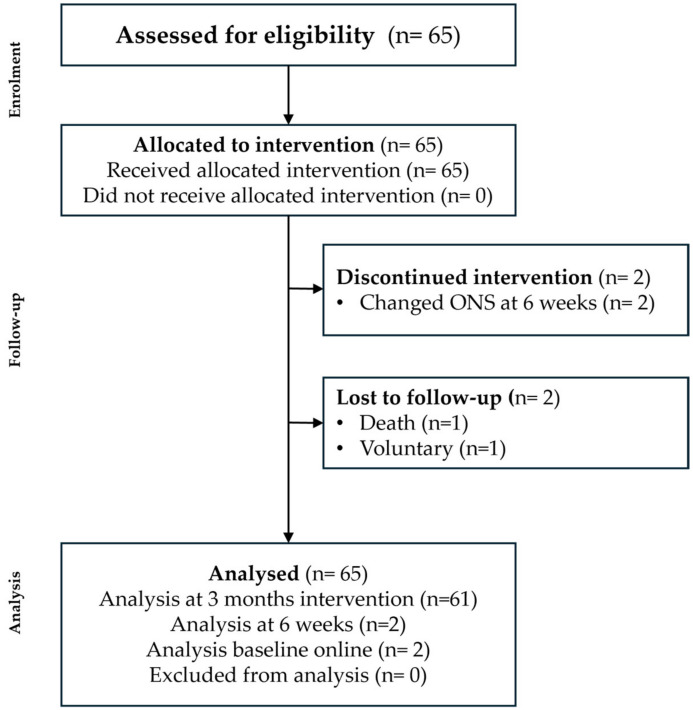
Flow chart of the included patients.

After the diagnosis, all patients initiated the nutritional intervention with the cHPHC-ONS. Among the participants, 35 (53.8%) patients consumed one bottle (200 mL), while 30 (46.2%) patients consumed two bottles (400 mL). During the three-month follow-up period, 61 (93.85%) patients continued consuming the concentrated oral nutritional supplement. However, two (3.1%) patients experienced gastrointestinal symptoms, specifically diarrhoea, during the latter half of the study. As a result, they switched to a different ONS, leading to a discontinuation of their participation in the study. However, they continue with this intervention for an additional six weeks with the intention to treat. Additionally, two patients discontinued the intervention (one patient died, and another patient stopped the ONS after one month) (Figure 3).

### 3.2. Evolution of Morphofunctional Assessment Variables

A significant reduction in percentage weight loss was observed, decreasing from −6.75% ± 7.50% at baseline to 0.5% ± 3.48 at three months (*p* < 0.01), although there was no significant weight gain (baseline: 51.39 ± 9.5 kg; 3 months: 51.62 ± 9.47 kg; *p* = 0.24). The ASMI showed a significant increase from 5.69 ± 0.94 kg/m^2^ at baseline to 6.34 ± 1.49 kg/m^2^ at three months (*p* < 0.01). Additionally, significant improvements were also noted in the RFMA (*p* = 0.03) and the grey level non-uniformity (*p* = 0.03). However, no significant changes were observed in other parameters (Table 2).

In patients aged ≥60 years, there was a significant increase in RFMA (*p* = 0.02), RFMT (*p* = 0.04), heterogeneity of grey level (*p* < 0.01), pennation angle (*p* = 0.02), and subcutaneous fat adipose tissue (*p* = 0.04), indicating positive changes. Additionally, this group of patients showed a statistically significant improvement in handgrip strength (*p* < 0.01), while no significant changes were observed in the BIA measurements (Table 3).

### 3.3. Changes in the Diagnosis of Malnutrition and Sarcopenia

**Figure 4:**
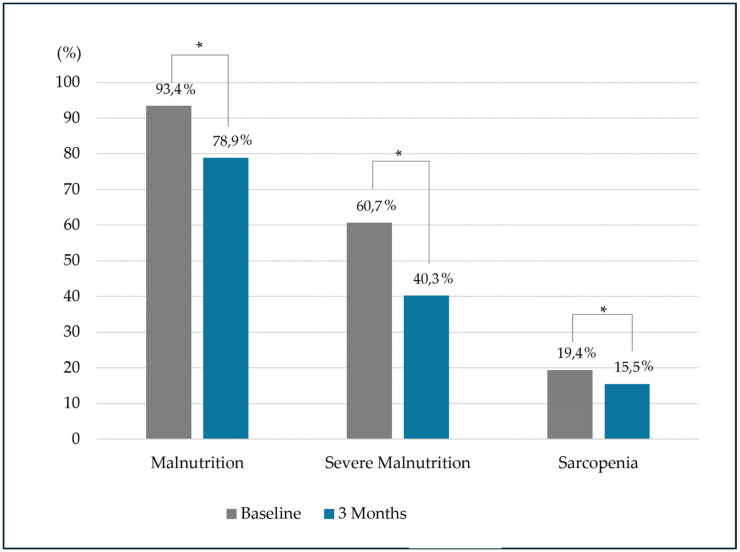
Differences in malnutrition and sarcopenia prevalence before and after the intervention. * denotes a statistically significant difference between baseline and follow-up period.

Over the three-month period, the intervention resulted in a remarkable and statistically significant reduction in the prevalence of malnutrition, dropping from 93.4% at baseline to 78.9% (*p* < 0.01), and in severe malnutrition, decreasing from 60.7% to 40.3% (*p* < 0.01; Figure 4).

**Figure 5:**
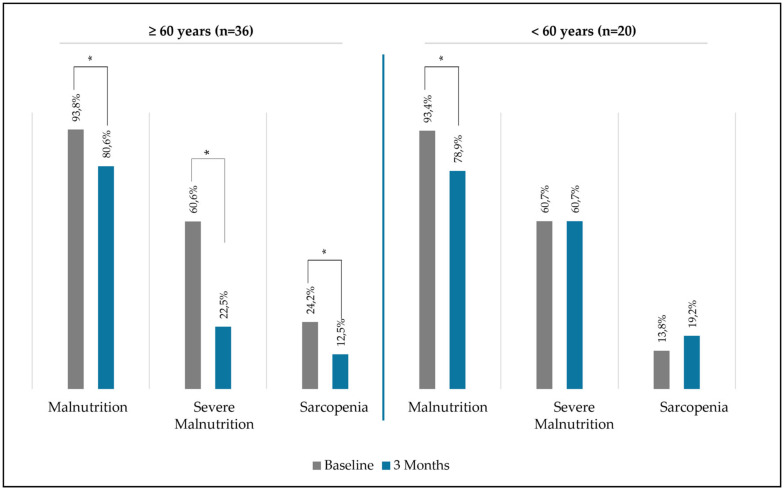
Differences in malnutrition and sarcopenia prevalence before and after the intervention in patients aged above and below 60 years. * *p*-value < 0.05.

Notably, patients aged ≥60 years showed a reduction in malnutrition from 93.8% to 80.6% (*p* < 0.01) and in severe malnutrition from 60.6% to 22% (*p* < 0.01) (Figure 5), while patients aged <60 years only showed a reduction in malnutrition prevalence (Figure 5).

The intervention resulted in a significant reduction in the prevalence of sarcopenia among all patients, decreasing from 19.4% at baseline to 15.5% after three months (*p* = 0.04; Figure 4). Remarkably, this improvement was only observed in patients aged ≥60 years (Figure 5). When we separated sarcopenia into its components, patients aged <60 years did not have differences in dinapenia prevalence before and after intervention (start: 17.2%; 3 months: 17.2%; *p* = 0.61). On the other hand, patients aged ≥60 years showed differences in prevalence before and after intervention (start: 36.1%; 3 months: 30.6%; *p* < 0.01).

### 3.4. Differences in Muscle US Parameters between ONS Consumption Amounts

**Figure 6:**
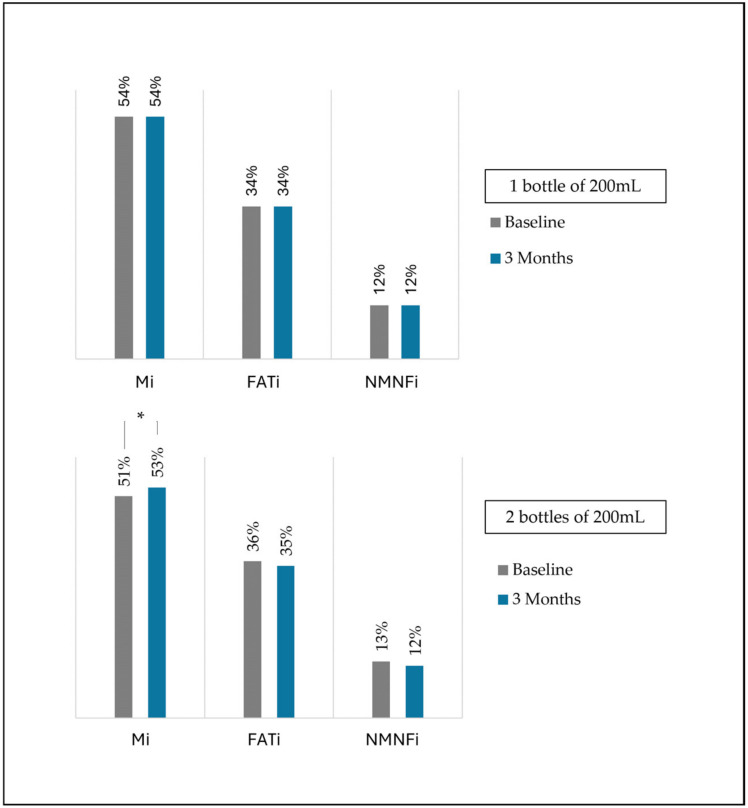
Differences in quality index measured in muscular ultrasonography between patients concerning oral nutritional supplement amount. Mi: muscle; FATi: fat; NMNFi: collagen, connective tissue, and fibrosis. * *p*-value < 0.05.

Patients who consumed two bottles experienced a statistically significant increase in estimated body cell mass compared to those who consumed one bottle (two bottles: +2.2% (IQR: −3.1% to +4.72%); one bottle: −2.2% (IQR: −16.40% to +0.86%); *p* < 0.01). Similarly, these patients also showed a statistically significant percentage increase in muscle quality index compared to those who consumed one bottle (two bottles: +4.24% (IQR: −6.24% to +13.75%); one bottle: −5.99% (IQR: −13.33% to +9.93%); *p* < 0.01). Although there was a non-significant increase in muscle quality index determined by multi-Otsu, a decrease in fat index was observed in patients who consumed two supplements (Figure 6). No significant differences were observed in the other parameters.

### 3.5. GI Tolerance and Compliance Rate

**Figure 7:**
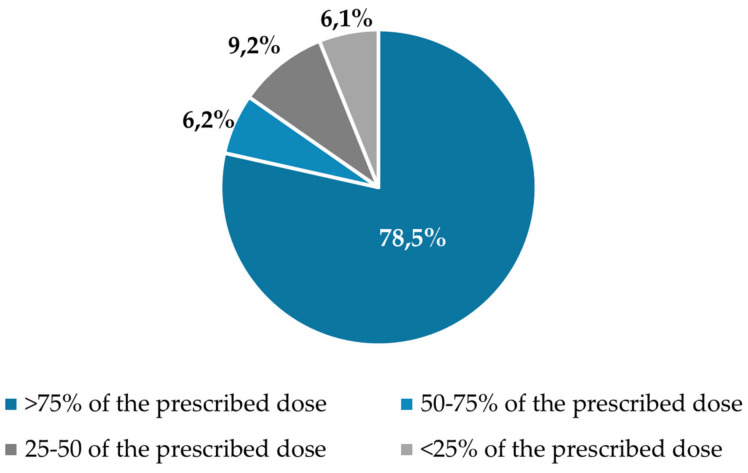
Rate of compliance to the prescribed dose.

The product exhibited good gastrointestinal (GI) tolerance. Only two patients (3.1%) experienced diarrhoea and needed to discontinue the intervention. They consumed <25% of the prescribed ONS. Compliance data are available for a total of 65 patients. A compliance rate of over 75% of the prescribed ONS was achieved by 78.5% of the sample (*n* = 51). Additionally, a compliance rate between 50% and 75% was achieved by 7.7% of the sample (*n* = 4), while a compliance rate between 25% and 50% was achieved by 6.2% of the sample (*n* = 9.2) and <25% in 6.2% of patients (*n* = 4) (Figure 7).

## 4. Discussion

In the present study, we evaluated a novel cHPHC-ONS containing high nutritional concentration (≥2.1 kcal/mL; 32g of protein per 200) and combining fast-acting and long-lasting, high-quality proteins (60% whey protein; 40% casein). This single nutritional formula addresses the specific needs of patients with DRM, who often suffer from decreased intake and require increased protein and caloric intake within a low nutrition volume. The high-protein content with a high load of branched-chain amino acids (BCAAs) is crucial for muscle protein synthesis and recovery for patients with DRM, who suffer from a high risk of sarcopenia. Additionally, the high energy and protein density of this supplement allows for the adequate adjustment of nutrient consumption within a manageable volume, which is vital for patient adherence.

We demonstrated that in patients at risk of malnutrition with low body weight, personalised nutritional intervention with cHPHC-ONS significantly prevented further weight loss. It led to improvements in ASMI, area, and echogenicity of the RF. Among patients aged ≥60 years, three months of cHPHC-ONS containing high-quality protein resulted in increased muscle area and thickness, as well as increased handgrip strength. Furthermore, the prevalence of malnutrition and sarcopenia decreased significantly three months after initiation of the personalised nutritional intervention with cHPHC-ONS.

It should be noted that our sample had a high percentage of oncological patients. This high prevalence of oncological patients was seen predominantly in patients aged ≥60 years. Ozorio et al. studied a sample of 885 cancer patients to assess DRM (median age = 61 years), which was highly prevalent among cancer patients [32]. The different distribution of pathologies related to age can influence the effect of personalised nutritional intervention on body composition. Patients with cancers have low-grade inflammation and an influence of treatment (surgery, radiotherapy, and chemotherapy), which can interfere with nutritional status and the response to medical nutrition support [33].

The comparison of body composition parameters between males and females showed higher baseline muscle mass and handgrip strength in males. The DRECO study had similar differences in terms of sex [7]; however, our sample had a higher prevalence of severe malnutrition. In terms of muscle quality, determined by the US for assessing echogenicity, the male patients showed higher values in percentage of muscle mass and lower values in percentage of fat intramuscular mass compared to female patients. These findings are similar to those of Van Doorn et al., which showed higher values of echogenicity (i.e., lower muscle mass) in healthy women than men [34].

We also found that the body composition parameters differed significantly between age groups. We found a lower phase angle in patients aged ≥60 years, which aligns with Mattiello et al.’s meta-analysis [35]. In the muscular US, older adults had low muscle mass (RFMA and RFMT) and less favourable muscle quality (higher percentage of muscle fat). These changes in muscle US were described by Rossi et al.; this study observed a low muscle mass and a higher echogenicity in older patients, with worse results in bedridden patients [36].

Body weight, BMI, and classic anthropometry parameters are unreliable variables used to monitor intervention effectiveness, especially in extreme weights (low and high) or pathologies such as cancer. These conditions are associated with changes in body composition related to inflammation and congestion [22]. Although, in our sample, we did not observe changes in body weight or BMI, the use of novel ONS prevented further weight loss, which is the primary objective of nutritional interventions in patients with DRM. These changes run in line with previous published studies. In 2022, in a study that analysed the effect of a diabetes-specific formula in a sample mainly composed of patients with oncologic pathology (68.3%), there was a reduction in weight loss percentage [37]. Another study from 2023, which mainly involved non-oncologic patients (82.1%), showed similar results [38]. The study of body composition through novel techniques, such as BIA and the muscular US, helped us monitor the changes with nutritional treatment.

BIA can help in assessing the body composition of patients with DRM. In our sample, we observed a decrease in phase angle, but there were no changes when we stratified our cohort according to the age groups. This lack of change across age groups may be due to varying causes of malnutrition that can affect body composition differently. A randomised clinical trial by Herrera A. et al. found no differences in phase angle among cancer patients who received a hypercaloric, hyperproteic formula with different types of protein [39]. In contrast, other studies have shown an increase in phase angle with long-term interventions. For example, Manikam et al. reported a numerical increase in phase angle in ovarian cancer patients undergoing chemotherapy after a six-month intervention [40]. Similarly, Cornejo-Pareja et al. demonstrated improvements in body composition parameters measured by BIA after six months, alongside decreased inflammatory burden. Notably, 60% of the patients in their study had been hospitalised due to oncological surgery [41]. Although BIA is beneficial, it is limited by the possible interference of the inflammation burden. Our results showed that, in patients with low inflammatory burden (patients with less than 60 years), there was an increase in estimated ASMI.

The effect of the cHPHC-ONS with high-quality protein was evident in the present study by the increased RF muscle mass (muscle area in total sample and muscle area and muscle thickness in patients over 60 years). These results are similar to those of a previous study of our group; in this study, we observed an increase in BMI-adjusted muscle area in men and in patients who consumed two bottles of an ONS enriched in leucine [38]. The study of Herrera et al. did not show differences in muscle mass assessed by the US in any group of investigation [39]. In older adults, the evidence of the effect of medical nutrition support on muscle US is scarce. Michel et al. did not observe muscle thickness changes in patients with progressive resistance training and increased protein intake [42]. Nevertheless, another study by Strasser et al. showed an increase in the thickness of all quadriceps muscles in a group of patients treated with resistance training and nutritional supplementation [43].

Sarcopenia is related to muscle mass and quality. Our study observed increased quality parameters of muscle US (pennation angle, grey level, and muscle thickness) and handgrip strength in older patients (over 60 years). Quality parameters of muscle US (pennation angle) and muscle strength in older patients increase in patients treated with protein supplementation with calcium β-hydroxy-β-methylbutyrate (CaHMB) and exercise training in some studies [44,45]. Increased handgrip strength has been observed in older patients with a high protein intake associated with exercise training. In our study, we increased protein intake and usual education in diet and exercise, which has been previously reported to improve muscle mass in older adults [46].

AI-based US imaging systems can help assess muscle mass and quality, avoiding human mistakes [23]. Quality parameters such as echogenicity of muscle and its relationship with echo architecture seem to be related to muscle function in patients with DRM [7], neurological patients [47] and older adults [48]. Previous reports demonstrated that the AI-based US assessment of RF muscle achieved high reliability and consistency [23]. These parameters can be used to evaluate the presence of sarcopenia and changes with nutritional treatment. Patients with lower age had more heterogeneous architecture and a higher percentage of muscle. In patients aged ≥60 years, intervention with cHPHC-ONS with high-quality protein led to a greater heterogeneity determined by grey level than those with younger age. In patients who consumed two bottles of ONS, we found an increase in the percentage of muscle mass relative to fat mass, as determined by the multi-thresholding algorithm. This finding aligns with previous research demonstrating that consuming two bottles of hypercaloric, leucine-enriched ONS increases muscle mass in patients undergoing medical nutritional treatment [38].

After the intervention, the patients had a lower prevalence of malnutrition and severe malnutrition, as assessed by GLIM criteria, and a reduction in sarcopenia prevalence. These findings were more consistent in patients older than 60 years. These results are similar to those of our studies with another hypercaloric hyperproteic supplementation, but these data were collected in a population with a lower rate of severe malnutrition [37].

The main strengths of our study are the use of novel ONS and advanced assessment techniques, such as muscular US and BIA, to assess the effectiveness of nutritional interventions in a population with severe DRM. To the best of our knowledge, limited evidence exists in real clinical practice regarding the effectiveness of this novel cHPHC-ONS, which contains a blend of fast-acting (60% whey protein and intrinsic leucine) and long-lasting (40% casein protein) high-quality proteins with all the spectrum of essential and BCAAs, to improve nutritional status and accelerate protein synthesis in these patients. During the three-month follow-up period, a high proportion of patients, 61 out of 65 (93.85%), demonstrated ongoing adherence to the prescribed therapeutic regimen by continuing to consume the supplement. Notably, only two patients (3.1%) experienced gastrointestinal symptoms (diarrhoea) and subsequently switched to an alternative ONS. Another strength is the use of an AI-based method to evaluate US imaging. However, the present study is limited by being a single-arm study with no control group; the main reason for this design is the scarcity of evidence with this type of ONS and the need to develop it. Our sample was also heterogeneous regarding underlying pathologies that caused malnutrition. Additionally, the inflammation burden can interfere with the results of phase angle and other components of body composition; however, this is a limitation in real-practice community-dwelling studies. Future studies are needed to evaluate the real effect of nutritional support. It is necessary to conduct randomised control trials with enteral formulas and to evaluate the effects with diagnostic tests that can be performed in routine clinical practice from a morphofunctional assessment point of view.

## 5. Conclusions

The present study supports the efficacy of the novel concentrated high-protein (32 g/200 mL), high-calorie (≥2.1 kcal/mL) oral nutritional supplement featuring a blend of fast- and slow-acting proteins (60% whey protein and 40% casein) in patients with disease-related malnutrition and sarcopenia. The results demonstrated a remarkable and statistically significant reduction in the prevalence of malnutrition, severe malnutrition, and sarcopenia, along with the prevention of further weight loss. Additionally, there was a notable increase in muscle mass, muscle quality, and overall muscle strength. Such findings underscore the effectiveness of personalised nutritional interventions using cHPHC-ONS in patients at high risk of severe malnutrition. Importantly, it was noted that individuals over 60 years of age, especially those who diligently adhered to the regimen of consuming two bottles daily, experienced the most significant benefits from the nutritional supplement. The implementation of morphofunctional assessment to monitor the nutritional status in our cohort allowed for a comprehensive understanding of changes in body composition beyond traditional anthropometric measures, further elucidating the impact of personalised medical nutrition therapy. While these outcomes are highly promising, further studies are needed to confirm our findings.

## Figures and Tables

**Table 1:** Differences in baseline morphofunctional variables by sex.

Variables (Unit)	Men (*n* = 24)	Women (*n* = 41)	*p*-Value
Age (years)	62.83 (16.06)	57.32 (17.94)	0.22
Anthropometry			
Weight (kg)	55.17 (10.78)	48.76 (7.77)	<0.01
BMI (kg/m^2^)	19.77 (3.42)	19.66 (3.57)	0.91
%Weight Loss (%)	9.91 (11.57)	6.44 (6.39)	0.13
Arm Circumference (cm)	23.35 (3.37)	22.22 (2.98)	0.16
Calf Circumference (cm)	30.12 (3.58)	30.33 (3.65)	0.82
Electrical Bioimpedanciometry			
Phase Angle (º)	5.34 (0.95)	4.88 (0.80)	0.04
Resistance/Height (ohm/m)	369.14 (68.95)	422.64 (78.65)	<0.01
Reactance/Height (ohm/m)	33.99 (6.38)	35.71 (7.93)	0.37
ASMI (kg/m2)	6.18 (0.89)	5.42 (0.81)	<0.01
BCMI (kg/m2)	8.09 (1.66)	7.22 (1.30)	0.03
Muscular Ultrasonography			
RFMA (cm^2^)	3.73 (1.05)	2.78 (0.92)	<0.01
RFMT (cm)	1.11 (0.23)	0.96 (0.23)	0.02
Mi	0.59 (0.11)	0.49 (0.08)	<0.01
FATi	0.30 (0.09)	0.34 (0.08)	<0.01
NMNFi	0.11 (0.05)	0.15 (0.05)	<0.01
Grey Level Non-Uniformity	2249 (594)	1989 (605)	0.09
Penation Angle (º)	4.31 (2.52)	4.67 (3.39)	0.65
SCFAT (cm)	0.48 (0.23)	0.78 (0.34)	<0.01
Muscle Function			
Handgrip Strength (kg)	28.61 (8.96)	18.77 (7.89)	<0.01
Biochemical Parameters			
Albumin (g/dl)	4.38 (0,37)	4.33 (039)	0.57
CRP/Prealbumin	0.40 (1.32)	0.11 (0.11)	0.29

BMI: Body Mass Index; ASMI: Appendicular Skeletal Muscle Index; BCMI: Body Cell Mass Index; RFMA: rectus femoris muscle area; RFMT: rectus femoris muscle thickness; Mi: Muscle to No Muscle Index; FATi: Fat to Muscle Index; NMNFi: No Muscle, No Fat to Muscle Index; SCFAT: subcutaneous fat; CRP: C-reactive protein.

**Table 2:** Morphofunctional variable changes: baseline vs. 3 months post-intervention.

Variables (Unit)	Baseline	3 Months	*p*-Value
Anthropometry			
Weight (kg)	51.39 (9.5)	51.62 (9.47)	0.24
BMI (kg/m^2^)	19.82 (3.49)	19.88 (3.48)	0.24
%Weight Loss (%)	−6.75 (7.50)	0.5 (3.48)	<0.01
Arm Circumference (cm)	22.72 (3.18)	22.71 (3.04)	0.93
Calf Circumference (cm)	30.25 (3.64)	30.46 (3.44)	0.33
Electrical Bioimpedanciometry			
Phase Angle (º)	5.07 (0.89)	4.95 (1.00)	0.04
Resistance/Height (ohm/m)	401.66 (81.36)	406.03 (83.42)	0.45
Reactance/Height (ohm/m)	35.35 (7.27)	34.96 (8.17)	0.49
ASMI (kg/m^2^)	5.69 (0.94)	6.34 (1.49)	<0.01
BCMI (kg/m^2^)	7.55 (1.52)	7.08 (1.71)	0.02
Muscular Ultrasonography			
RFMA (cm^2^)	3.14 (1.08)	3.36 (1.19)	0.03
RFMT (cm)	1.02 (0.25)	1.05 (0.27)	0.18
TDRF (cm)	3.49 (3.18–3.89)	3.54 (3.13–3.84)	0.17
Mi	0.53 (0.10)	0.53 (0.10)	0.67
FATi	0.35 (0.08)	0.35 (0.07)	0.92
NMNFi	0.12 (0.04)	0.12 (0.04)	0.41
Grey Level Non-Uniformity	2063 (609)	2215 (678)	0.03
Penation Angle (º)	4.63 (3.10)	4.84 (2.90)	0.64
SCFAT (cm)	0.69 (0.32)	0.72 (0.35)	0.14
Muscle FunctioN			
Handgrip Strength (kg)	22.12 (9.52)	23.16 (9.69)	0.07
Biochemical Parameters			
Albumin (g/dl)	4.35 (0.38)	4.29 (0.41)	0.24
CRP/Prealbumin	0.05 (0.04–0.13)	0.06 (0.04–0.89)	0.89

BMI: Body Mass Index; ASMI: Appendicular Skeletal Muscle Index; BCMI: Body Cell Mass Index; RFMA: rectus femoris muscle area; RFMT: rectus femoris muscle thickness; TDRF: transversal diameter rectus femoris; Mi: Muscle to No Muscle Index; FATi: Fat to Muscle Index; NMNFi: No Muscle, No Fat to Muscle Index; SCFAT: subcutaneous fat; CRP: C-reactive protein.

**Table 3:** Age-related changes in morphofunctional variables: baseline vs. 3 months post-intervention.

Variables (Unit)	≥60 Years (*n* = 36)			<60 Years (*n* = 29)		
	Baseline	3 Months	*p*	Baseline	3 Months	*p*
Anthropometry						
Weight (kg)	54.25 (10.25)	54.39 (10.15)	0.71	47.94 (7.28)	48.25 (7.44)	0.29
BMI (kg/m^2^)	21.14 (3.51)	21.14 (3.54)	0.99	18.22 (2.78)	18.34 (2.75)	0.26
%Weight Loss (%)	−7.66 (8.4)	2.75 (13.49)	<0.01	−5.86 (6.24)	0.67 (3.23)	<0.01
Arm Circumference (cm)	23.13 (3.48)	23.06 (3.16)	0.82	22.23 (2.75)	22.28 (2.89)	0.79
Calf Circumference (cm)	30.51 (3.61)	30.75 (3.27)	0.52	29.94 (3.71)	30.10 (3.66)	0.26
Electrical Bioimpedanciometry						
Phase Angle (º)	4.78 (0,72)	4.62 (0,94)	0.06	5.44 (0.95)	5.36 (0.96)	0.38
Resistance/Height (ohm/m)	390.51 (63.02)	390.75 (76.55)	0.97	415.29 (98.89)	424.70 (88.99)	0.25
Reactance/Height (ohm/m)	32.44 (6.56)	31.47 (7.27)	0.18	38.92 (6.56)	39.24 (7.19)	0.71
ASMI (kg/m^2^)	5.72 (0.79)	6.15 (1.55)	0.09	5.65 (1.11)	6.56 (1.43)	0.01
BCMI (kg/m^2^)	7.33 (1.29)	6.88 (1.77)	0.07	7.77 (1.71)	7.27 (1.66)	0.13
Muscular Ultrasonography						
RFMA (cm^2^)	2.66 (0.89)	3.03 (1.21)	0.02	3.69 (1.02)	3.73 (1.09)	0.69
RFMT (cm)	0.88 (0.18)	0.95 (0.23)	0.04	1.16 (0.23)	1.16 (0.28)	0.89
TDRF (cm)	3.49 (3.01–3.96)	3.48 (3.13–3.90)	0.23	3.46 (3.04–3.77)	3.55 (3.12–3.77)	0.67
Mi	0.49 (0.10)	0.49 (0.07)	0.54	0.56 (0.09)	0.58 (0.10)	0.21
FATi	0.36 (0.06)	0.37 (0.05)	0.20	0.33 (0.07)	0.31 (0.72)	0.10
NMNFi	0.15 (0.04)	0.14 (0.04)	0.32	0.10 (0.03)	0.10 (0.04)	0.93
Grey Level	1811 (553)	2125 (804)	<0.01	2352 (547)	2318 (491)	0.63
Penation Angle (º)	3.38 (2.14)	4.58 (2.68)	0.02	6.04 (3.44)	5.14 (3.16)	0.26
SCFAT (cm)	0.65 (0.27)	0.71 (0.29)	0.04	0.73 (0.36)	0.73 (0.41)	0.95
Muscle Function						
Handgrip Strength (kg)	21.06 (10.81)	22.69 (11.22)	<0.01	23.37 (7.74)	23.73 (7.66)	0.73

BMI: Body Mass Index; ASMI: Appendicular Skeletal Muscle Index; BCMI: Body Cell Mass Index; RFMA: rectus femoris muscle area; RFMT: rectus femoris muscle thickness; TDRF: transversal diameter rectus femoris; Mi: Muscle to No Muscle Index; FATi: Fat to Muscle Index; NMNFi: No Muscle, No Fat to Muscle Index; SCFAT: subcutaneous fat.

## Data Availability

The data supporting this study’s findings are available from the corresponding author upon reasonable request.

## Supplementary Materials

The following supporting information can be downloaded at https://www.mdpi.com/article/10.3390/nu16183136/s1, Table S1: Composition of high-protein energy-dense oral nutrition supplement (ONS) used as an intervention. Table S2: Differences in morphofunctional variables in function of age (more or less than 60 years).
