# Supplementary material for: Effectiveness of High-Protein Energy-Dense Oral Supplements on Patients with Malnutrition Using Morphofunctional Assessment with AI-Assisted Muscle Ultrasonography: A Real-World One-Arm Study
Source: Nutrients. 2024 Sep 17;16(18):3136. doi: 10.3390/nu16183136 (PMC11435358; doi:10.3390/nu16183136)
Supplement: Supplementary file 1 [file nutrients-16-03136-s001.zip › nutrients-3181185-supplementary.pdf]

Supplementary Table S1. Nutritional Composition per serving (200mL) of the concentrated high-protein, high-calorie Oral Nutrition Supplement used as an intervention.

**Nutritional component (Unit)**	**1 bottle of 200mL**
**Energy (kcal / kJ))**	420/1760
**Proteins** (g)	32<br>(60% Whey protein/40% Casein)
L-Leucine (g)	3,6
**Fats** (g)	21
Saturated (g)	2.2
Monounsaturated (g)	12
Polyunsaturated (g)	5.2
**Carbohydrates** (g)	25.8
Sugars (g)	17
**Vitamins**	
Vitamin A (mcg)	250
Vitamin D (mcg)	3.6
Vitamin E (mg)	3.6
Vitamin K (mcg)	26
Vitamin C (mg)	40
Thiamin (mg)	1.3
Riboflavin (mg)	0.72
Vitamin B_6 (mg)	0.6
Niacin (mg)	1
Folic Acid (mg)	100
Vitamin B_{12} (mg)	1.5
Pantothenic acid (mg)	1.3
Biotin (mg)	10
**Minerals**	
Sodium (mg)	300
Potassium (mg)	460
Chloride (mg)	260
Calcium (mg)	430
Phosphorus (mg)	360
Magnesium (mg)	60
Iron (mg)	3.8
Zinc (mg)	3.6
Manganese (mg)	0.40
Copper (mg)	0.60
Iodine (mcg)	50
Selenium (mcg)	20
Chromium (mcg)	16
Molybdenum (mcg)	22

Supplementary Table S2: Differences in Morphofunctional Variables by Age: ≥60 years old vs.<60 years old

Variables (Unit)	≥60 years (n=36)	<60 years (n=29)	p-value
**Age (years)**	71.31 (8.4)	44.52 (13.73)	<0.01
**Gender (H/M)**	61.1%/38.9%	65.5%/34.5%	0.13
**ANTHROPOMETRY**			
**Weight (kg)**	53.69 (10.35)	47.93 (7.14)	0.01
**BMI (kg/m^2)**	20.85 (3.63)	18.28 (2.76)	<0.01
**%Weight loss (%)**	9.21 (10.37)	6.04 (6.20)	0.16
**Arm Circumference (cm)**	22.93 (3.48)	22.27 (2.71)	0.41
**Calf Circumference (cm)**	30.41 (3.57)	30.05 (3.69)	0.69
**ELECTRICAL BIOIMPEDANCIOMETRY**			
**Phase Angle (°)**	4.75 (0.73)	5.43 (0.92)	<0.01
**Resistance/Height (ohm/m)**	391.88 (61.96)	416.54 (95.61)	0.21
**Reactance/Height (ohm/m)**	32.37 (6.44)	38.42 (7.20)	<0.01
**ASMI (kg/m2)**	5.73 (0.75)	5.65 (1.09)	0.74
**BCMI (kg/m2)**	7.28 (1.28)	7.78 (1.65)	0.21
**MUSCULAR ULTRASONOGRAPHY**			
**RFMA (cm^2)**	2.74 (0,90)	3.61 (1.08)	<0.01
**RFMT (cm)**	0.91 (0.19)	1.16 (0.23)	<0.01
**Mi**	0.50 (0.10)	0.56 (0.09)	<0.01
**FATi**	0.36 (0.07)	0.29 (0.08)	<0.01
**NMNFi**	0.14 (0.06)	0.12 (0.05)	<0.01
**GLNU**	1898 (585)	2317 (569)	<0.01
**Pennation Angle (°)**	3.32 (2.20)	6.04 (3.38)	<0.01
**SCFat (cm)**	0.61 (0.29)	0.75 (0.37)	0.09
**MUSCLE FUNCTION**			
**Handgrip Strength (kg)**	21.41 (10.91)	23.48 (7.59)	0.39
**BIOCHEMICAL PARAMETERS**			
**Albumin (g/dl)**	4.4 (0.40)	4.28 (0.36)	0.23
**CRP (mg/dl)**	1.1 (1-4.97)	1 (1-1.95)	0.02
**CRP/Prealbumin**	0.34 (1.11)	0.08 (0.07)	0.75

BMI: Body Mass Index; ASMI: Appendicular Skeletal Muscle Index; SMI: Skeletal Muscle Index; BCMI: Body Cell Mass Index; RFMA: rectus femoris muscle cross sectional area; RFMT: rectus femoris muscle thickness; Mi: Muscle to no Muscle Index; FATi: Fat to muscle index; NMNFi: No Muscle-No fat-to muscle index; SCFat: Subcutaneous Fat Thickness; GLNU: measures the variability of gray-level intensity values in the image, with a lower value indicating more homogeneity in intensity values; CRP: C-Reactive Protein
